# Cardiac amyloidosis mimicking severe aortic valve stenosis – a case report demonstrating diagnostic pitfalls and role of dobutamine stress echocardiography

**DOI:** 10.1186/s12872-017-0519-0

**Published:** 2017-03-22

**Authors:** Tim Salinger, Kai Hu, Dan Liu, Sebastian Herrmann, Kristina Lorenz, Georg Ertl, Peter Nordbeck

**Affiliations:** 1 Department of Internal Medicine I, University Hospital Würzburg, Würzburg, Germany; 2 Comprehensive Heart Failure Center (CHFC), University of Würzburg, Würzburg, Germany; 3 Leibniz-Institut für Analytische Wissenschaften – ISAS, University Duisburg-Essen, Dortmund, Germany; 4 Medizinische Klinik und Poliklinik I – Kardiologie, Universitätsklinikum Würzburg, Oberdürrbacher Str. 6, 97080 Würzburg, Germany

**Keywords:** Aortic valve stenosis (AS), Cardiac amyloidosis, Dobutamine stress echocardiography, Low-gradient AS, Pseudo-severe AS, Case report

## Abstract

**Background:**

Aortic valve stenosis is a common finding diagnosed with high sensitivity in transthoracic echocardiography, but the examiner often finds himself confronted with uncertain results in patients with moderate pressure gradients and concomitant systolic heart failure. While patients with true-severe low-gradient aortic valve stenosis with either reduced or preserved left ventricular systolic function are primarily candidates for valve replacement, there is a relevant proportion of patients with pseudo-severe aortic valve stenosis anticipated not to benefit but actually rather deteriorate by interventional therapy or surgery.

**Case presentation:**

In this article we present a case report of a male patient with pseudo-severe aortic valve stenosis due to cardiac amyloidosis highlighting the diagnostic schedule. The patient underwent stress echocardiography because of discrepant findings in transthoracic echocardiography and cardiac catheterization regarding the severity of aortic valve stenosis. After evaluation of the results, it became clear that he had a need for optimum heart failure medication and implantation of a cardiac resynchronization therapy defibrillator.

**Conclusion:**

Due to the pitfalls in conventional as well as invasive diagnostics at rest, Stress echocardiography should be considered part of the standard optimum diagnostic spectrum in all unclear or borderline cases in order to confirm the correct diagnosis and constitute optimal therapy.

## Background

Stenosis of the aortic valve (AS) is the most common valvular disease in elderly persons today. Previous studies have reported prevalence for moderate or severe AS of 5–8% in a population older than 74 years [1, 2]. While current guidelines recommend conservative medical treatment in patients with mild and moderate AS [3], severe AS is highly recommended for surgical treatment especially in symptomatic patients, because of the poor prognosis of this disease entity in patients under conservative therapy [3, 4]. In elderly patients ≥80 years with severe AS, 3-year mortality has been described to be approximately 40% [5].

The diagnostic cornerstone in the differentiation between moderate and severe AS is transthoracic echocardiography. By convenient use of this widely available, non-invasive, and cost saving imaging technique, the experienced echocardiographer is able to determine the transvalvular pressure gradients and calculate the valve area with high diagnostic accuracy in most patients [3]. However, diagnostic accuracy can be considerably diminished in certain patients. This particularly refers to patients with a decreased left-ventricular-ejection-fraction (LVEF), occasionally leading to low transvalvular pressure gradients even if the opening area of the valve is indeed severely restricted, as e.g. confirmed by transesophageal echocardiography, usually referred to as low-gradient AS [6]. On the other hand, an echocardiographically confirmed severely diminished aortic valve opening area in this patient group might also be functional due to the profoundly reduced LV stroke volume leading to incomplete opening of the aortic valve, usually referred to as pseudo-severe AS [7]. Some patients may also have low aortic valve pressure gradient and severe AS, but preserved LVEF [8]. This so called paradoxical low-flow, low gradient AS is particularly difficult to diagnose and standard diagnostics often lead to false results and inadequate therapy [9]. While conclusive differentiation of the before mentioned disease entities is highly important for therapy planning in this patient group with high procedural risk during aortic valve replacement (AVR), it often represents a diagnostic dilemma for the cardiologist particularly in case of combined valvular diseases or myocardial comorbidities since even invasive measurements are often inconclusive in such cases.

## Case presentation

An 82 year old male patient was referred to our cardiological department for treatment of aortic valve stenosis. The patient was in substantially reduced general condition with dyspnoe on insignificant loads (NYHA III) and edemas of hands and feet. Physical examination revealed a grade 3/6 systolic ejection murmur with radiation to the carotid arteries. There had been multiple hospitalizations in the past 6 month because of recurrent cardiac decompensations. Previously known comorbidities were chronic heart failure for unknown reason, atrial fibrillation, pulmonary hypertension and chronic obstructive pulmonary disease. Cardiovascular risk factors were arterial hypertension, diabetes, and former nicotine abuse. Biochemical tests revealed a moderately decreased glomerular filtration rate (54 ml/min/1.73qm), slightly increased creatinine (1.35 mg/dl) and increased high sensitivity troponin (98.5 pg/ml). NT-proBNP was 5473 pg/ml.

**Fig. 1:**
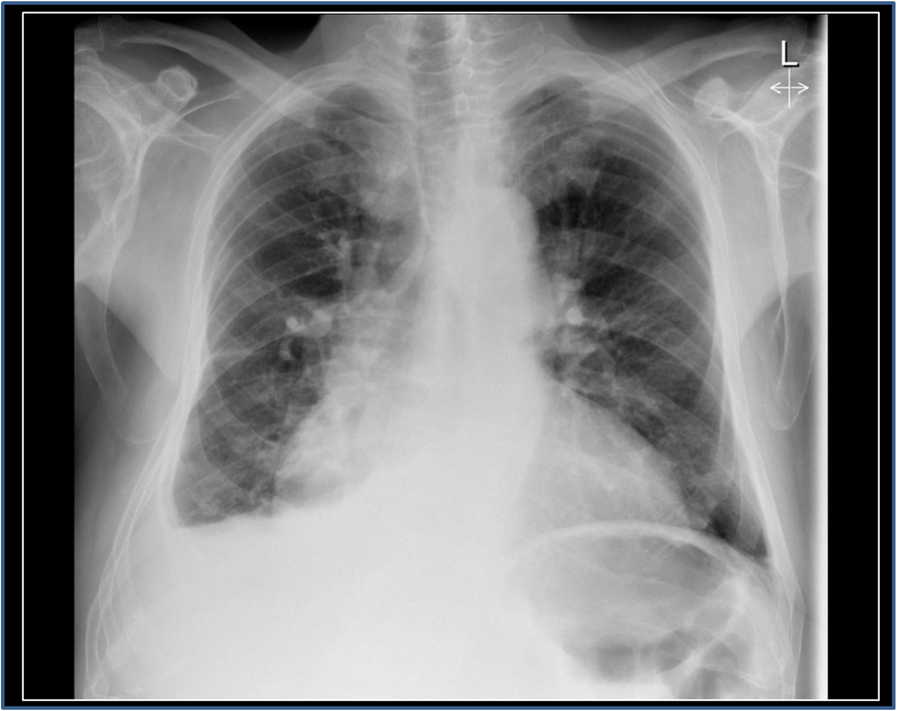
Chest X-ray depicting a global enlarged heart, chronic pulmonary congestion, and right-sided pleural effusion

**Fig. 2:**
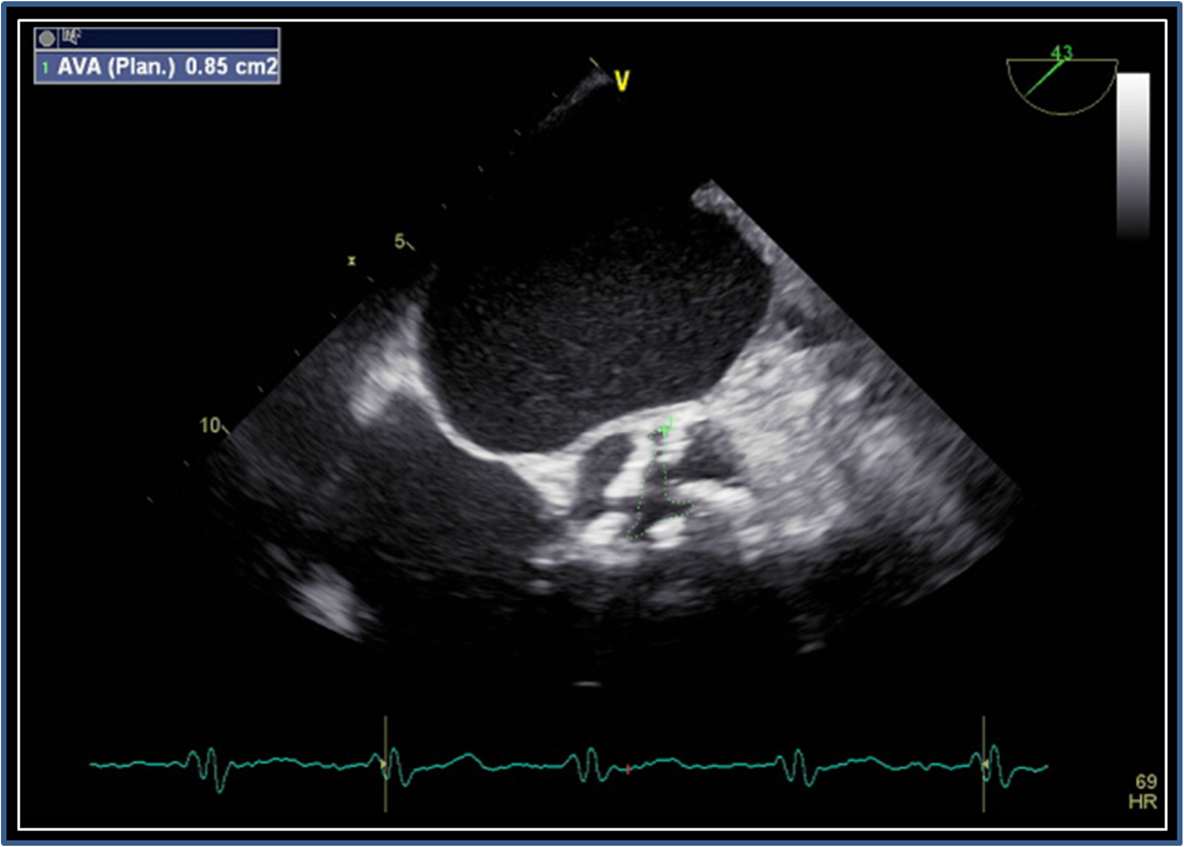
Morphologic aspect of the aortic valve in transesophageal echocardiography. Aortic valve area is calculated as 0.85 cm^2^ by planimetry

The patient underwent transthoracic echocardiography for staging severity of the aortic valve stenosis, which revealed borderline results (peak aortic jet velocity 2.8 m/s, mean aortic jet velocity 2.1 m/s, peak aortic pressure gradient 31 mmHG, mean aortic pressure gradient 19 mmHG, calculated aortic valve area 0.9 cm^2^). Global left ventricular systolic function was highly reduced (LVEF Simpson 4CH = 29%), stroke volume index was 24 ml/qm. Global longitudinal strain was -7.3%. Apical sparing, which could be a sign for cardiac amyloidosis [10] was not present. Transthoracic echocardiography also revealed diastolic dysfunction (e = 1.01 m/s; e’ septal = 0.03 m/s; e’lateral = 0.04 m/s; s’septal 0.02 m/s; s’lateral = 0.03 m/s, e/e’ = 28.9). The left atrium was moderately, and the right atrium profoundly enlarged (35 cm^2^ each, left atrial volume index 67.8 ml/qm). Chest X-ray showed a global enlarged heart, chronic pulmonary congestion, and right-sided pleural effusion (Fig. 1). Coronary angiography showed no significant coronary artery stenosis. Laevocardiography confirmed a highly reduced LVEF and a massively increased left ventricular end-diastolic pressure (33 mmHg), Right heart catheterization showed a normal cardiac output at rest (cardiac index 3.1 l/min*m^2^) and post-capillary pulmonary hypertension (pulmonary artery mean pressure 44 mmHG, max 66 mmHg, pulmonary capillary wedge pressure 35 mmHG). Invasively measured aortic valve gradients were 25/16 (max/mean) mmHG and the aortic valve opening area was calculated as 1.9 cm^2^. In transesophageal echocardiography, the opening area of the aortic valve was planimetrically determined as 0.8 cm^2^, again indicating severe AS (Fig. 2).

**Fig. 3:**
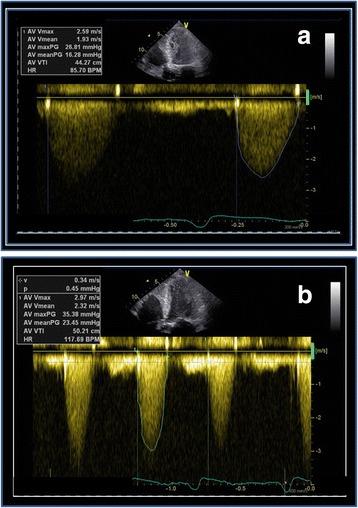
**a** Stress echocardiography at rest conditions. AV mean pressure is calculated as 16 mmHg. **b** Under stress conditions (20 gamma dobutamine), AV mean pressure did not exceed 23 mmHg

Due to discrepant findings in echocardiography and cardiac catheterization regarding the severity of aortic valve stenosis, low-dose dobutamine stress echocardiography was performed (Fig. 3). At rest conditions, there was global hypokinesia with an ejection fraction of 33%. Aortic valve (AV) mean pressure was 16 mmHg referring to a calculated aortic valve opening area of 0,7 cm^2^. Under peak dobutamine stress (20 mcg/kg/min), LVEF increased to 47% mean and mean AV pressure gradient to 23 mmHg. Left ventricular outflow tract velocity increased more than aortic valve velocity (LVOT/AV 0.2 at rest vs. 0.25 under stress) referring to an enlarged opening area of the aortic valve under stress conditions from 0.7 cm^2^ to 1.1 cm^2^. Stroke volume increased by 61.3% to 50 ml. On the basis of these diagnostic findings, AS was classified as pseudo-severe due to systolic dysfunction of the left ventricle with preserved contractile reserve, indicating a non-valvular reason for heart-failure and no indication for valve replacement.

**Fig. 4:**
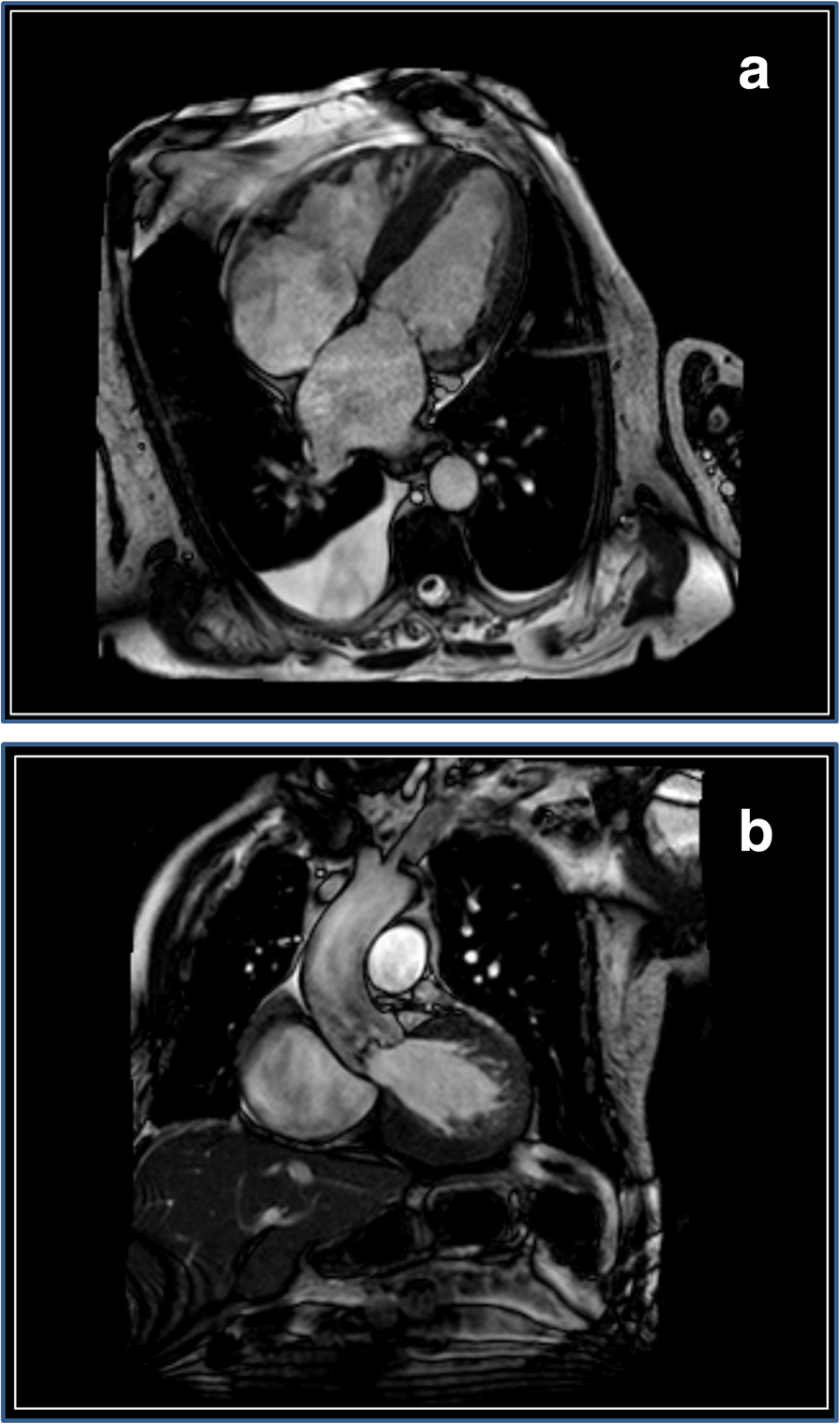
**a** Native MRI, 4-chamber view, showing enlarged atria und left ventricular hypertrophy (LA 40 mm^2^; RA 41 mm^2^, lateral wall thickness 14 mm; septal wall thickness 16 mm) as signs of cardiac amyloidosis. **b** Morphologic aspect of the left ventricular outflow tract and aortic valve in MRI

**Fig. 5:**
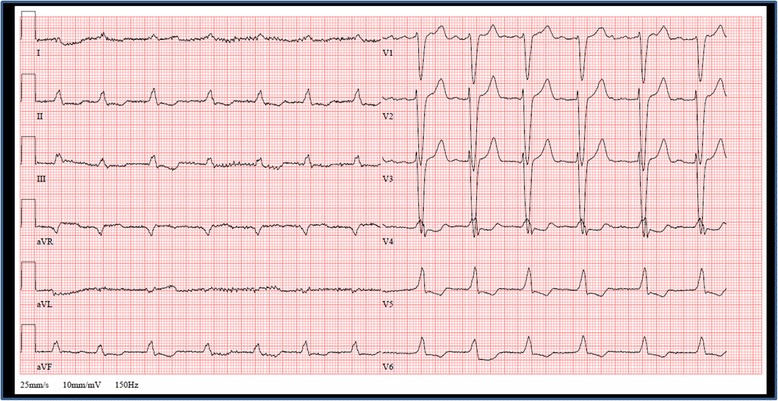
ECG at admission shows atrial fibrillation at 78 bpm, left bundle branch block and signs of left ventricular hypertrophy

Since the origin of the highly reduced LVEF was still unclear, cardiac MRI was performed, confirming global hypokinesia with a LVEF of 35% and revealing reduced myocardium-to-blood contrast, raising suspicion for presence of a cardiomyopathy (Fig. 4). An endomyocardial biopsy from the intraventricular septum in the right ventricular outflow tract was done, revealing multifocal interstitial transthyretin- related cardiac amyloidosis (ATTR). Because of a newly developed complete left bundle branch block in the ECG (Fig. 5) and the severely reduced LVEF, implantation of a cardiac resynchronization therapy defibrillator (CRT-D), was performed on top of optimum heart failure medication. Regrettably, at short- and medium-term follow-up echocardiography, LVEF did not show relevant improvement, although no signs of cardiac dyssynchrony could be found. However, NYHA improved to NYHA II and in the following 2-year period after device implantation, no more hospitalizations were required. Status of AS in follow-up transthoracic echocardiography 1 year later did not differ from baseline.

## Discussion and conclusions

This clinical case highlights the potential value of dobutamine stress echocardiography in anticipated low-grade true-severe AS. After the initial echocardiography showing low LVEF, a mean valvular gradient <40 mmHg and a calculated valve area <1 cm^2^, the patient was suspected to have low-flow, low-gradient aortic stenosis [3]. This disease entity would normally lead to valve replacement because of the potential benefits in terms of symptoms and survival gained from this procedure [3, 11], but should also be assessed very carefully when planning valve replacement. The American College of Cardiology recommends AVR for “symptomatic patients with severe AS and decreased systolic opening in calcified or congenitally stenotic aortic valves and an aortic velocity of >4.0 m/s or greater, or a mean pressure gradient of 40 mmHg or higher and symptoms of heart failure” [11]. However, in particular patients without contractile reserve have been shown to have exceedingly high operative mortality [12]. Differentiation between patients with low-flow, low-gradient stenosis and those with pseudo-severe stenosis can be very difficult in clinical practice since echocardiographic characteristics might differ only marginally at rest between both groups. Dobutamine stress echocardiography was first used as early as 1995 to distinguish these two subgroups. Based on the results from this study, it was already speculated that a broader use of this method might be clinically useful [13]. In 2001 it was first shown that clinical management of individuals with aortic stenosis and left ventricular dysfunction as well as low or intermediate valve gradients can indeed be improved by dobutamine stress echocardiography, because not only the distinction between patients with severe and non-severe aortic stenosis is improved, but this has indeed relevant implications on clinical outcome [14]. True-severe low-flow, low-gradient stenosis patients primarily benefit from AVR, since the valve itself is the main problem, whereas the primary pathology in pseudo-severe stenosis is located in the myocardium, requiring intensified conservative or specialized treatment based on the underlying disease [15].

In the present case, aortic stenosis of our patient could be clearly classified pseudo-severe by low dose dobutamine stress echocardiography. In the respective guidelines of the European Society of Cardiology, this procedure is not well established, but mentioned as potentially useful for the aforesaid diagnostic indication [3]. It is a widely-used, non-invasive method that can be done not only in-hospital but also in ambulatory patient care. Examination time is actually short and procedural risk has shown to be low [13]. Therefore, the net benefit greatly outweighs potential patient risks, justifying the investment in cases similar to the one described above.

Following stress-echocardiography, we performed cardiac MRI to reveal the origin of high-grade systolic dysfunction in our patient, revealing typical signs of an infiltrative cardiomyopathy such as late gadolinium enhancement [16] and dilated atria as well as a thickened septum and global left ventricular hypertrophy. High sensitive troponin was checked and turned out to be elevated. This serum biomarker has been shown to be a useful but non-specific diagnostic tool in various hereditary or secondary cardiomyopathies such as e.g. Sarcoidosis or Anderson-Fabry disease [17, 18]. In the described case, the final diagnosis ultimately clarifying the etiology of the pathological myocardial findings was made by endomyocardial biopsy from the right ventricle. Histological findings clearly revealed cardiac amyloidosis.

Amyloidosis is a systemic disease which can affect every organ, but especially cardiac involvement is a major determinant in patients prognosis [19]. In general, amyloidosis is considered a rare disease. However, there is a high probability of under-diagnosis particularly in elderly patients, where specific symptoms can be easily misinterpreted as an expression of advanced aging processes. Different studies have also shown that Amyloidosis and AS may occur together in a significant number of patients [20–23]. Today, several diagnostic tools are available to rule out whether a patient is indeed affected by cardiac amyloidosis, including the ECG, serum biomarkers, echocardiography, MRI and nuclear medicine imaging techniques [24, 25]. In cases where additional advanced imaging techniques are not available, standard echocardiographic examinations can be expanded by speckle tracking analysis of left ventricular (systolic) function [26]. However, in order to come to a definite diagnosis, current guidelines recommend endomyocardial biopsy [27]. Guidelines for therapy also recommend intensified heart failure therapy in respective patients with mainly systolic dysfunction. However, the impact of device therapy such as CRT-D on top of medical therapy on symptoms and prognosis in cardiac amyloidosis is currently not well investigated.

The presented case highlights some important aspects and pitfalls in diagnosis and treatment of elderly patients with aortic valve stenosis transferred to hospital for aortic valve replacement. While the natural history of low-gradient aortic valve stenosis and concept of optimum therapy including valve replacement is generally better understood today, great care has to be taken not to exaggerate these findings to patients with pseudo-severe aortic valve stenosis. Stress echocardiography should be considered part of the standard optimum diagnostic spectrum in all unclear or borderline cases in order to confirm the correct diagnosis and constitute optimal therapy.

## Abbreviations

AS: Aortic stenosis
AV: Aortic valve
AVR: Aortic valve replacement
CRT-D: Cardiac resynchronization therapy defibrillator
LVEF: Left- ventricular- ejection- fraction
